# Prevalence of paucibacillary cases of leprosy in Brazil: a 20-year systematic review and meta-analysis

**DOI:** 10.3389/fmed.2024.1401685

**Published:** 2024-11-13

**Authors:** Bruna Eduarda Brito Gonçalves, André Matheus Porto Raiol, Ana Vitória Cruz Brito, Marcos Jessé Abrahão Silva, Daniele Melo Sardinha, Karla Valéria Batista Lima, Luana Nepomuceno Gondim Costa Lima

**Affiliations:** ^1^Department of Nurse, University of Amazon (UNAMA), Ananindeua, Brazil; ^2^Center for Biological and Health Sciences (CCBS), State University of Pará (UEPA), Belém, Brazil; ^3^Molecular Biology Laboratory, Bacteriology and Mycology Section (SABMI), Evandro Chagas Institute (IEC), Ananindeua, Brazil

**Keywords:** Brazil, leprosy, paucibacillary, *Mycobacterium leprae*, epidemiology

## Abstract

**Introduction:**

Leprosy is a chronic infectious disease caused by the agent *Mycobacterium leprae*, characterized by its high disabling power. Data points to Brazil being the second country with the highest number of cases in the world, behind only India, representing a major challenge for public health. This work aims to determine the prevalence of paucibacillary (PB) cases in relation to leprosy cases in Brazil, using data published in the literature.

**Methods:**

This is a systematic review and meta-analysis carried out with studies from the last 20 years, being developed based on the Preferred Reporting Items for Systematic Review and Meta-analyzes (PRISMA).The search was carried out in the databases: PUBMED, SciELO, LILACS (via VHL)and Science Direct in October 2023, using the following descriptors (((“Brazil” [Mesh]) AND (“Leprosy, paucibacillary” [Mesh])) AND “Epidemiology” [Mesh]), in English, Portuguese and Spanish. Original studies of the analytical case–control, cohort, cross-sectional, epidemiological types were selected, as well as articles with satisfactory information for numerical extraction with separate data on the paucibacillary and multibacillary clinical forms. The methodological quality assessment followed the JBI critical appraisal checklist. Statistical analysis was performed using the Comprehensive Meta-Analyses-CMA software, version 3.0 (Biostat, Engewood, NJ, United States).

**Results:**

The meta-analysis of the 48 studies obtained a paucibacillary prevalence rate in Brazil of 50.5% or 0.505 (95% CI = 0.502–0.509).The differences in the analyzes were statistically significant (Q-value 4302.681;df 47; I 98.905), with a high heterogeneity value evidenced by I^2^ (98.905). This analysis demonstrated that the frequency in the Midwest region was the highest and the South region was the lowest (21.4%). Begg’s (Kendall Tau *p* = 0.35) and Egger’s tests (*p* = 0.20) were performed, in which no high publication bias was noted. Subgroup analysis indicated that paucibacillary cases varied from region to region, with the Midwest region having the highest prevalence and the South region having the lowest.

**Conclusion:**

The results stand out significantly for the research gaps that investigate PB cases, requiring more research aimed at investigating the paucibacillary clinical form that can contribute to the early diagnosis of leprosy.

**Systematic review registration:**

PROSPERO code: CRD42024514106.

## 1 Introduction

Leprosy is a chronic infectious disease caused by the agents *Mycobacterium leprae* and recently discovered *Mycobacterium lepromatosis*, characterized by its high disabling power that can present itself in different clinical forms with dermatological and neurological signs and symptoms, with affinity for phagocytic cells, such as macrophages and Schwann cells. It has exogenous transmission through the upper airways, occurring through close and prolonged contact with the untreated bacilliferous patient (1–3).

According to the World Health Organization (WHO), leprosy remains a persistent challenge to public health. Despite significant global efforts and established strategies, indicators continue to point to the persistence of morbidity. The current strategy adopted by the WHO aims to interrupt transmission and achieve the elimination of indigenous cases, aiming to achieve a zero-incidence rate for leprosy [3]. Brazil in recent years occupies the second position in terms of leprosy cases in the world, behind only India. According to the 2022 epidemiological bulletin from the Ministry of Health (MS), between 2016 and 2020, 155,359 new cases were registered, with 2020 being the year with the lowest global detection rate, which is associated with a reduction in diagnoses due to overload in health services during the COVID-19 pandemic in which health services were overloaded (4).

The persistence of the spread of the disease is associated with several issues, with late diagnosis highlighted as one of the main factors, as it favors the spread between contacts and contributes to the advancement of the condition, resulting in the development of disabilities that compromise nervous function (5, 6). Inadequate diagnosis, especially when differentiating leprosy cases, reveals a hidden presence of the disease, compromising the accuracy of officially reported epidemiological data (7). Furthermore, delay in diagnosis becomes an alarming factor that contributes to the emergence of disabilities, which consequently cause numerous biopsychosocial and economic impacts for both the individual and society (8). Territorial expansion and population increase are correlated with the advancement of leprosy, being determinant for the high detection rates of the disease (9). In this context, early identification combined with timely administration of treatment can reduce the chances of progression and worsening of leprosy (10).

The manifestation of clinical forms of leprosy is linked to the host’s immune response. The WHO classifies patients based on the number of lesions and/or smear results, with additional categorization as multibacillary in cases of uncertainty. Paucibacillary (PB) individuals have up to five skin lesions on sputum smear microscopy, while Multibacillary (MB) individuals have six or more lesions and/or a positive result on sputum smear microscopy. The Madrid classification considers clinical, microscopic, bacteriological, immunological and histological aspects, dividing leprosy into indeterminate, tuberculoid, dimorphic or borderline and lepromatous forms. The WHO defines as paucibacillary those with indeterminate, tuberculoid or borderline-tuberculoid forms, and a bacilloscopic index of less than five lesions in all sites surveyed (11).

The paucibacillary classification of leprosy refers to the initial phase of the disease, characterizing individuals with less symptomatic or asymptomatic forms. This category is marked by the limited presence of skin and neurological lesions, with a low bacterial load, making these individuals less likely to be significant sources of transmission. Diagnosis faces challenges due to the similarity of clinical manifestations with several other dermatological conditions, such as pityriasis *alba*, granulomatous *rosacea*, granuloma *nulla*, among others (12). The detection of leprosy in the PB clinical form faces challenges due to several factors, including the lack of confirmatory laboratory methods. Although the effectiveness of real-time PCR (qPCR) has been demonstrated with respect to specificity and sensitivity in several studies, more comprehensive investigations are needed to evaluate its applicability in clinical practice (13).

Considering the challenging detection of the paucibacillary form in individuals with leprosy, the clinical evolution of the disease tends to worsen and progress to the multibacillary form. Due to this scenario, confirmed cases of paucibacillary leprosy are less frequent in Brazil compared to multibacillary cases (14). However, the prevalence of cases, especially in endemic regions, is predominantly associated with intense local transmission. Given this scenario, in endemic regions, targeting surveillance initiatives to prioritize early diagnosis, treatment and assessment of contacts become essential to interrupt the transmission chain (15).

Systematic analyzes provide a high degree of evidence in clinical epidemiology, playing a crucial role in guiding decisions in both public and private spheres. Meta-analysis, in turn, is a statistical approach that synthesizes the results of two or more independent studies that address the same research question, consolidating the summarized data into a comprehensive statistical measure (16). In this sense, we intend to carry out a systematic review and meta-analysis study to understand the epidemiological situation of paucibacillary (PB) cases of leprosy in Brazil, providing a quantitative assessment of these factors.

This research can help identify patterns of geographic variation and distribution, highlighting the regions with the highest incidences, contributing to the development of specific strategies in the most affected regions, and may be fundamental for directing public health policies, guiding prevention strategies and early diagnosis. In addition to contributing significantly to the scientific literature, providing a synthesis of the available evidence from the last 20 years regarding the prevalence of PB cases, providing important insights to identify areas in which more research is needed related to the topic, as well as providing information for researchers, healthcare professionals, health and policy development. In this sense, the research question guiding this study is: what is the prevalence of paucibacillary cases in the last 20 years in Brazil?

## 2 Materials and methods

### 2.1 Type of study, protocol, and registration

This is a systematic review and meta-analysis with the objective of determining the prevalence of paucibacillary (PB) cases in relation to leprosy cases in Brazil, using data published in the literature. Following the recommendations of the Preferred Report Items of a Systematic Review and Meta-Analyses (PRISMA) 2020 Statement (17). This ratio meta-analysis was carried out in this study for two correlated investigations: (I) To identify the prevalence of paucibacillary cases in Brazil and (II) to determine the prevalence of cases by region. The article was registered on the PROSPERO platform with the code CRD42024514106.

### 2.2 Research strategy

Regarding the purpose of this review, the formulation of the research question was based on the acronym POT (Population, Outcome and Type of study), being derived from the acronym PICO (Population, Intervention, Comparator and Outcome). Which consisted of the following question: “What is the prevalence of paucibacillary cases in the last 20 years in Brazil?” The anagram for its formation was composed of “P” for problem (paucibacillary cases), “O” for outcome (prevalence of cases in Brazil) and T for type of study (original and epidemiological studies).

The databases investigated for the search were PubMed (accessed October 17, 2023),^1^ SciELO (accessed on October 17, 2023),^2^ Science Direct (accessed October 17, 2023),^3^ and LILACS (via VHL) (accessed on October 17, 2023),^4^ as these databases in Latin America have weight and contain the majority of Brazilian publications.

The health descriptors available in Medical Subject Headings (MeSH) were used. The search strategy that was developed for the databases had the following descriptors: (((“Brazil” [Mesh]) AND (“Leprosy, paucibacillary” [Mesh])) AND “Epidemiology” [Mesh]). And after the search, filters related to eligibility criteria such as full texts, languages and publication period were applied.

### 2.3 Eligibility criteria

Original studies published in Portuguese, English or Spanish, from January 2003 to October 2023, focused on inclusion criteria of particular types of research designs (descriptive and analytical observational studies) of the types case–control, cohort, cross-sectional, epidemiological, and with sufficient data for numerical extraction with separate data from clinical forms (paucibacillary and multibacillary) were eligible. For the research provided to this meta-analysis, only observational studies were selected as the selection technique since bias can arise in original studies due to defects in the included study’s design, which tend to distort the size or direction of relationships in the data (18).

Brief/short communications, letters to the editor, clinical trials, reviews, editorials, articles unavailable in their complete form, articles outside the stipulated time frame, review articles and studies outside the inclusion criteria mentioned above were excluded.

### 2.4 Study selection and data extraction

Data was collected from all databases on October 17, 2023. The selection process was carried out in two phases. In the first phase, two researchers (BEBG and MJAS) independently screened the titles and abstracts to identify eligible studies using online software (Rayyan, Qatar Computing Research Institute). Then, in the second phase, three researchers (BEBG, AMPR and AVCB) read the eligible studies in full. Any disagreements between the three reviewers regarding the inclusion, exclusion and/or extraction of research data were resolved by re-evaluation and agreement between their two supervisors (DMS and MJAS) to make the final decision. For the qualitative analysis, only studies that met the minimum eligibility criteria were included.

For data extraction and synthesis, all extracted data were stored in Microsoft Excel file format and the following information was extracted from each study: Authors and year of publication; Title; Kind of study; Data base; Purpose of the article; Location, State and Brazilian Region analyzed in the appropriate added study and period; Total population analyzed; Operational classification with the occurrence of multibacillary cases (n, %) and paucibacillary cases (n; %); and JBI score.

### 2.5 Assessment of methodological quality

Quality assessment was performed by two researchers (DMS and BEBG) using the Joanna Briggs Institute (JBI) Critical Appraisal Checklist for Analytical Cross-Sectional Studies (0–8), the JBI Checklist for Case Studies- control (0–10), and the JBI Checklist for Cohort Studies (0–11). Only when the conditioned answer was “Yes” were the scores for completing the checklist questions taken into account. A third researcher (MJAS) provided an opinion in case of disagreement (19).

### 2.6 Statistical analysis

The Comprehensive Meta-Analyses—CMA software, version 4.0 (Biostat, Engewood, NJ, United States), was used to perform the statistical analyzes of the meta-analysis. The analysis was conducted using a randomized effect approach, considering a *p* < 0.05 to indicate statistical significance. Heterogeneity was assessed using the I-square test (I^2^). Furthermore, the Egger and Begg classification correlation tests were applied, together with the use of a funnel plot, to investigate possible publication bias, being considered statistically significant when *p* < 0.05. Subgroup analysis was carried out according to Brazilian regions and meta-regression was used to explore the origin of the identified heterogeneity.

## 3 Results

### 3.1 Selection of the studies

**Figure 1:**
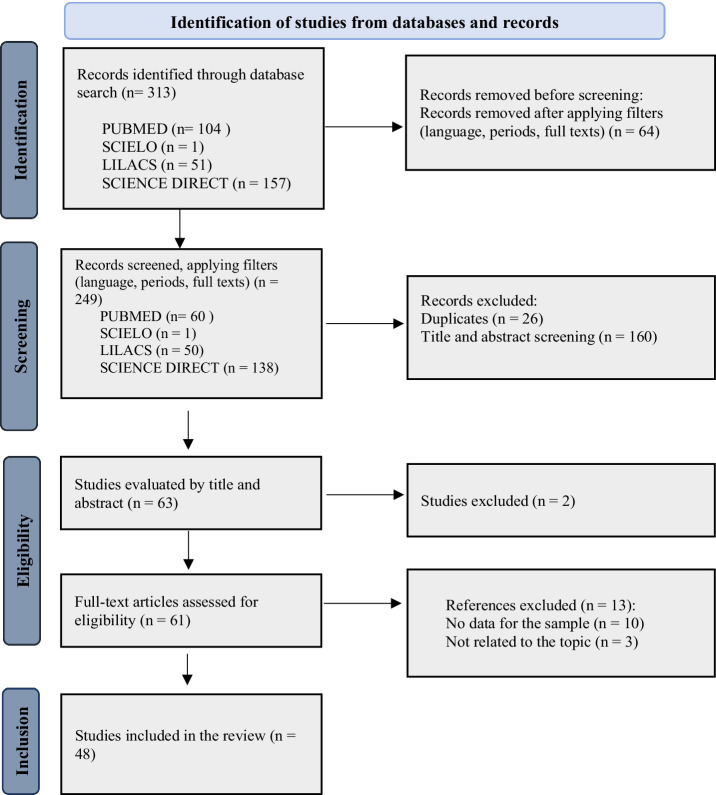
PRISMA flow diagram (2020) and selection of included studies.

After searching health databases, 313 publications were tracked: 104 PubMed, 1 SciELO, 51 LILACS, 157 Science Direct. Of these results, 249 remained after applying the filters (languages, period of studies and complete texts), screening of relevant titles was carried out (studies with data from paucibacillary cases), 148 studies were excluded, these being because they were not related to the study objective, as they did not provide data to compose the sample and 26 duplicate studies were also excluded. In this sense, we evaluated 75 complete articles in full, for data extraction and composing the sample (Supplementary material S1). After reading and analysis, 27 studies were excluded and 48 were eligible to compose the sample and included in this systematic review (Figure 1).

### 3.2 Characterization of included studies

**Figure 2:**
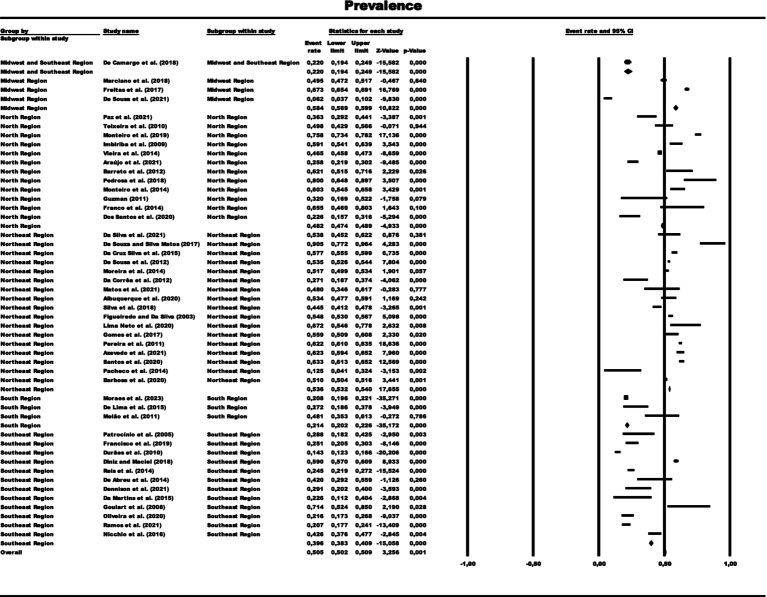
Forest graph representing the prevalence of paucibacillary cases by total sample grouped by subgroups of Brazilian regions. The size of each square corresponds to the weight of the related research in the meta-analysis, representing the Odds Ratio (OR) of each study on the map. The 95% confidence intervals (CI) for OR from each study are shown as horizontal lines. Numbers in bold indicate the overall OR and 95% CI, as well as the total frequency of cases and controls.

A total of 48 studies were included in the final analysis. The publication period of the studies was a 20-year segment covering the period from 2003 to 2023. The most frequent language was English, followed by Portuguese. The Northeast (*n* = 17; 35.42%), North (*n* = 12; 25%) and Southeast (*n* = 12; 25%) regions were the most represented in relation to the studies developed (Table 1; Figure 2).

**Table 1:** Characteristics of the included studies.

No.	Authors (year)	Title	Kind of study	Data base	Purpose of the article	Location, state, and Brazilian region/period	Total analyzed	Classification				JBI score
								MB	n(%)	PB	n(%)	
1	Paz et al. (2021)	Association of the polymorphism of the vitamin D receptor gene (VDR) with the risk of leprosy in the Brazilian Amazon (62).	Case–control	PUBMED	To evaluate the association of SNPs FokI (rs2228570), TaqI (rs731236), ApaI (rs7975232) of the VDR gene with leprosy.	Rondom do Pará, Cúrialândia, Goiânia and cities of Redenção, state of Pará, Region	157	100	24.60	**57**	14.10	8/10
2	Teixeira et al. (2010)	Epidemiological and clinical characteristics of leprosy reactions in paucibacillary and multibacillary individuals treated at two leprosy reference centers in the city of Recife, state of Pernambuco (63)	Cross-sectional	SciELO, LILACS	Describe the epidemiological and clinical characteristics of leprosy reactions in paucibacillary and multibacillary individuals.	Amazon, Brazil, North	201	101	50.20	**100**	49.80	8/8
3	Da Silva et al. (2021)	Epidemiological scenario of leprosy and differences by sex (64)	Ecological time series study	LILACS	Describe the epidemiological scenario of leprosy according to clinical and demographic characteristics among female and male individuals.	Municipality of Paulo Afonso, in Bahia, Northeast (2014–2017)	130 (new cases)	58	44.60	**70**	53.90	9/9
4	De Souza and Silva Matos (2017)	Characterization of leprosy in children under 15 years in an important municipality of north-eastern Brazil (53)	Observational, cross-sectional and descriptive	LILACS	To identify the family, clinical and epidemiological characteristics of individuals under 15 years of age who were diagnosed with leprosy in the city of Juazeiro-BA.	Juazeiro-BA/Brazil, (2012–2014)	42 (NEW CASES)	4	9.50	**38**	90.50	6/8
5	Patrocínio et al. (2005)	Detection of *Mycobacterium leprae* in nasal mucosa biopsies by the polymerase chain reaction (65)	Cross-sectional	ScienceDirect, PUBMED	To evaluate the clinical application of PCR for detecting *M. leprae* in lower nasal turbinate biopsies in untreated leprosy patients and their contacts.	Uberlândia, MG, Brazil, Southeast	52	37	71.20	**15**	28.80	6/8
6	Marciano et al. (2018)	Epidemiological and geographical characterization of leprosy in a Brazilian hyperendemic municipality (8)	Retrospective, observational and descriptive ecological design	SciELO, PUBMED	To identify the distribution pattern of leprosy in a hyperendemic municipality in Brazil and determine its relationship with the clinical-epidemiological situation over 11 years.	Rondonópolis, state of Mato Grosso/Brazil, mid-west (2000–2010)	1832	921	50.4	**906**	49.6	9/9
7	Da Cruz Silva et al. (2015)	Epidemiological aspects of leprosy in Juazeiro-BA, from 2002 to 2012 (66)	Descriptive, cross-sectional	SciELO, PUBMED	To analyze the epidemiology of leprosy in Juazeiro-BA, from 2002 to 2012.	Juazeiro-BA/Brazil, Northeast (2002–2012)	1916	810	42.30	**1,106**	57.70	9/9
8	Moraes et al. (2023)	Epidemiological characteristics of leprosy from 2000 to 2019 in a state with low endemicity in southern Brazil (28)	Retrospective observational	ScienceDirect, SciELO, PUBMED	Characterize the epidemiological profile of leprosy in the state of Rio Grande do Sul from 2000 to 2019.	Rio Grande do Sul/Brazil, South (2000–2019)	4,233	3,318	79	**882**	21	9/9
9	De Sousa et al. (2012)	Epidemiological Profile of Leprosy in the Brazilian state of Piauí between 2003 and 2008 (67)	Descriptive	SciELO	To evaluate the clinical and epidemiological profile of patients with leprosy between 2003 and 2008 in the Brazilian state of Piauí and analyze the detection and prevalence rates in the general population and in the child population under 15 years of age.	Piauí/Brazil, northeast (2003–2008)	12,238	5,632	46.02	**6,551**	53.53	6/8
10	Moreira et al. (2014)	Epidemiological situation of leprosy in Salvador from 2001 to 2009 (68)	Retrospective cross-sectional	SciELO	To analyze the epidemiological situation, the detection rate and evaluate the clinical and epidemiological profile of leprosy in Salvador, in the period 2001–2009.	Salvador-BA/Brazil, Northeast (2001–2009)	3,226	1,535	47.6	**1,667**	51.7	6/8
11	Da Corrêa et al. (2012)	Epidemiological, clinical, and operational aspects of leprosy patients assisted at a referral service in the state of Maranhão, Brazil (23)	Cross-sectional	SciELO, LILACS, PUBMED	Describe the epidemiological, clinical and operational aspects of leprosy patients.	São Luis - MA, northeast (2008–2009)	85	62	72.90	**23**	27.10	9/9
12	Matos et al. (2021)	Epidemiological, neurofunctional profile and prevalence of factors associated with the occurrence of physical disabilities due to leprosy in a reference center in Northeast Brazil: a sectional study (69)	Cross-sectional	SciELO, PUBMED	To describe the epidemiological and neurofunctional profile, as well as the prevalence of factors associated with the occurrence of physical disabilities due to leprosy in a reference center in Northeast Brazil.	Juazeiro-BA/Brazil, Northeast (JAN-JUN, 2018)	50	26	52	**24**	48	6/8
13	Albuquerque et al. (2020)	Epidemiological, temporal and spatial dynamics of leprosy in a municipality in northeastern Brazil (2008–2017): an ecological study (70)	Ecological	SciELO, LILACS, PUBMED	To analyze the epidemiological, temporal and spatial dynamics of leprosy in a municipality in the Northeast of Brazil.	Arapiraca-AL/Brazil, Northeast (2008–2017)	292	136	46.60	**156**	53.40	8/9
14	Francisco et al. (2019)	Estimation of the hidden prevalence of leprosy in a municipality in the interior of the State of São Paulo (9)	Descriptive, epidemiological	LILACS	Analyze the leprosy situation in a municipality in the interior of the State of São Paulo in relation to the global situation, and calculate the hidden prevalence	Municipality in the interior of the State of São Paulo, (2006–2016)	295 cases reported	221	74.10	**74**	25.90	9/9
15	Durães et al. (2010)	Epidemiological study of 107 familial leprosy outbreaks in the city of Duque de Caxias - Rio de Janeiro, Brazil (71)	Descriptive, epidemiological	SciELO, LILACS	Analyze epidemiological data on the variables: sex, age, years of study, degree of kinship with the IC and type of residential contact (intra-domestic or per-domestic) with the IC in 107 leprosy families.	Duque de Caxias-RJ, (1998–2002)	1,040	176	17	**149**	14	8/9
16	Silva et al. (2018)	Factors associated with leprosy in a municipality of the Pre-Amazon region, state of Maranhão, Brazil (72)	Descriptive, analytics	SciELO, LILACS, PUBMED	Evaluate the clinical and epidemiological characteristics of the disease to reduce the detection rate of new cases by 2015.	Buriticupu, Maranhão/Brazil, Northeast (2003–2015)	879	488	55.50	**391**	44.50	6/8
17	Monteiro et al. (2019)	Hansen’s disease in children under 15 years old in the state of Tocantins, Brazil, 2001–2012: epidemiological patterns and temporal trends (73)	Descriptive, Epidemiological	PUBMED, LILACS, SciELO	To describe the epidemiological characteristics and temporal trends of leprosy indicators in children under 15 years of age in Tocantins between 2001–2012.	State of Tocantins/Brazil, (2001–2012)	1,225	296	24.20	**929**	75.80	9/9
18	Imbiriba et al. (2009)	Leprosy in indigenous populations of Amazonas, Brazil: an epidemiological study in the municipalities of Autazes, Eirunepé and São Gabriel da Cachoeira (2000 to 2005) (74)	Descriptive, epidemiological	SciELO,	Description and analysis of the epidemiological characteristics of leprosy notifications in the municipalities of Autazes, Eirunepé and São Gabriel da Cachoeira, comparing findings between indigenous and non-indigenous people, according to variables of interest. Cases reported in SINAN from 2000 to 2005 were analyzed.	Municipalities of Autazes, Eirunepé and São Gabriel da Cachoeira, Amazonas, Northern Region (2000 to 2005)	386	157	40.70	**228**	59.30	9/9
19	Vieira et al. (2014)	Leprosy in Rondônia: incidence and characteristics of reported cases, 2001 to 2012 (75)	Descriptive	SciELO, LILACS	Describe and analyze the incidence of leprosy and the characteristics of cases reported in the State of Rondônia, Brazil, from 2001 to 2012	Rondônia, Northern Region (2001–2012)	15,648	8,353	54.4	**7,282**	46.6	9/9
20	Araújo et al. (2021)	Hanseniasis in the municipality of Western Amazon (Acre, Brazil): are we far from the goal of the World Health Organization?: Hansen and Western Amazon (76)	Ecological, temporal	ScienceDirect, SciELO, PUBMED	Analyze the epidemiological profile and trends of leprosy from 2005 to 2018, in addition to the clinical characteristics of the patients.	Hospital of Sanitary Dermatology, Cruzeiro do Sul, Acre, North (2005 to 2018)	**422**	307	73.9	**109**	25.8	9/9
21	Barreto et al. (2012)	High rates of undiagnosed leprosy and subclinical infection among school children in the Amazon Region (59)	Cross-sectional	PUBMED, SciELO	Determine the prevalence of subclinical infection (defined in this study as seropositivity for IgM anti-PGL-I without clinical signs or symptoms of leprosy) and the prevalence of undiagnosed leprosy among CS in selected municipalities in the state. SHOVEL.	Altamira - PA; Briefs -PA; Castanhal - PA; Marituba - PA; Oriximiná - PA; Paragominas - PA; Parauapebas - PA; Redemption - PA. North (2009–2011)	87	33	37.9	**54**	62.1	9/9
22	Figueiredo and Da Silva (2003)	[Increase in leprosy detection rates in São Luís, Maranhão, Brazil, from 1993 to 1998: is the endemic expanding?] (77)	Epidemiological, descriptive	PUBMED, SciELO	Write and analyze aspects of the detection of new cases of leprosy in São Luís from 1993 to 1998	São Luis - MA, northeast (1993–1998)	2,796	1,263	45.17	**1,533**	54.83	9/9
23	Pedrosa et al. (2018)	Leprosy among schoolchildren in the Amazon region: A cross-sectional study of active search and possible source of infection by contact tracing (54)	Cross-sectional	PUBMED	Determine the detection rate of previously undiagnosed leprosy cases among schoolchildren under 15 years of age living in Manaus, Amazonas, Brazil, and their possible source of infection through contact tracing	Manaus, Amazonas, North (2014–2016)	40	8	20	**32**	80	6/8
24	De Lima et al. (2015)	Leprosy in a University Hospital in Southern Brazil (55)	Descriptive, retrospective, cross-sectional	SciELO, PUBMED	Evaluate the epidemiological profile of patients monitored at a University Hospital	Hospital de Clinicas UFPR, Paraná, south (2005–2010)	81	57	70.32	**22**	26.56	5/8
25	Lima Neto et al. (2020)	Leprosy in children under 15 years of age in a municipality in northeastern Brazil: evolutionary aspects from 2003 to 2015 (56)	Descriptive	SciELO, PUBMED	Evaluate the epidemiological characteristics of the disease in children under 15 years of age	Buriticupu, Maranhão, northeast (2003–2015)	61	20	32.8	**41**	67.2	8/10
26	Diniz and Maciel (2018)	Leprosy: clinical and epidemiological study in patients over 60 years in Espírito Santo State – Brazil (78)	Observational, descriptive and retrospective	SciELO, LILACS	Describe the epidemiology and clinical aspects of leprosy cases in individuals over 60 years of age	Espirito Santo, (2001–2011), Southeast Region	2,510	1,030	41	**1,480**	59	9/9
27	Monteiro et al. (2014)	Limited activity and social participation after hospital discharge from leprosy treatment in a hyperendemic area in northern Brazil (79)	Cross-sectional	SciELO, PUBMED	Characterize the limitation of activity and social participation and its correlation with disabilities and/or disabilities in individuals after discharge from multidrug therapy for leprosy.	Araguaína, Tocantins, North (2004–2009)	282	112	39.7	**170**	60.3	8/8
28	Reis et al. (2014)	*Mycobacterium leprae* DNA in peripheral blood may indicate a bacilli migration route and high-risk for leprosy onset (80)	Cross-sectional	ScienceDirect, PUBMED	To evaluate the presence of *M. leprae* DNA in peripheral blood samples from leprosy patients and their household contacts	CREDESH, Minas Gerais, southeast	1,026	775	75.5	**251**	24.5	6/8
29	De Abreu et al. (2014)	*Mycobacterium leprae* is identified in the oral mucosa from paucibacillary and multibacillary leprosy patients (81)	Cross-sectional, retrospective	ScienceDirect, PUBMED	Investigate the presence of *M. leprae*, through IHC and PCR, in the oral mucosa of leprosy patients and compare the results of PB and MB patients	Institute of Tropical Medicine, Faculty of Medicine, University of São Paulo, Southeast Region	50	29	58.00	**21**	42	6/8
30	Dennison et al. (2021)	*Mycobacterium leprae*–helminth co-infections and vitamin D deficiency as potential risk factors for leprosy: A case–control study in south-eastern Brazil (82)	Case–control	ScienceDirect, PUBMED	Investigate possible associations between leprosy, helminth infections and micronutrients	Governador Valadares and Mantena, Minas Gerais, southeast (2016–2018)	79	53	67	**23**	29.1	8/10
31	Da Martins et al. (2015)	Nasal mucosa study of leprosy contacts with positive serology for the phenolic glycolipid 1 antigen (83)	Cross-sectional, prospective	ScienceDirect, SciELO, PUBMED	Identify specific and early leprosy lesions through endoscopic, bacilloscopy, histopathological examinations and real-time polymerase chain reaction of the nasal cavity mucosa in household and household contacts with positive serology for the phenolic glycolipid antigen 1.	Duque de Caxias, Rio de Janeiro, Southeast Region (2003–2006)	31	24	77.4	**7**	22.6	6/8
32	Guzman (2011)	Clinical-epidemiological profile of leprosy cases reported in the municipality of Assis Brasil, Acre, from 2003 to 2010 (84)	Epidemiological, descriptive	LILACS	To describe the epidemiological characteristics of leprosy in the municipality of Assis Brasil, in Acre, from 2003 to 2010.	Assis Brasi, Acre (2003–2010)	25	17	68	**8**	32	9/9
33	Franco et al. (2014)	Profile of cases and risk factors for leprosy, in children under 15 years of age, in a hyperendemic municipality in the northern region of Brazil (85)	Ecological, longitudinal	LILACS	Demonstrate the temporal pattern of leprosy, clinical aspects and contact relationships, in children under 15 years of age, in an area of a former leprosy colony in the North of Brazil,	Igarapé Açu, Pará, north (2003–2013)	29	10	34.5	**19**	65.5	9/9
34	Gomes et al. (2017)	Epidemiological profile of leprosy in a hyperendemic municipality in northeastern Brazil (86)	Epidemiological, cross-sectional and descriptive	SciELO, LILACS	Analyze the epidemiological profile of leprosy in Floriano, Piauí.	Floriano, Piauí. Northeast (2009–2013)	388	170	43.81	**217**	55.93	9/9
35	Pereira et al. (2011)	Epidemiological profile of leprosy in the municipality of Teresina, in the period 2001–2008 (87)	Descriptive	SciELO, LILACS	Describe the epidemiological profile of the municipality of Teresina from 2001–2008.	municipality of Teresina, northeast (2001–2008)	5,921	2,231	38	**3,685**	62	9/9
36	Melão et al. (2011)	Epidemiological profile of patients with leprosy in the extreme south of Santa Catarina, from 2001 to 2007 (88)	Cross-sectional and retrospective study	SciELO, LILACS	To know the profile of patients with leprosy in the municipalities of the Association of Municipalities of the Carboniferous Region (AMREC), from 01/01/2001 to 12/31/2007	Association of Municipalities of the Carboniferous Region, Santa Catarina, South (2001–2007)	54	27	50	**26**	48.15	6/8
37	Azevedo et al. (2021)	Epidemiological profile and spatial distribution of leprosy in Paulo Afonso, Bahia (89)	Descriptive epidemiological	SciELO, LILACS	analyze the epidemiological characteristics and distribution of new leprosy cases in the population of Paulo Afonso, Bahia, between 2000 and 2015.	Paulo Afonso, Bahia, Northeast (2000 and 2015).	1,069	403	37.7	**666**	62.30	8/9
38	Santos et al. (2020)	Epidemiological profile and leprosy trend in children under 15 years of age (27)	Quantitative, cross-sectional and descriptive	SciELO, LILACS	To evaluate the epidemiological characteristics and trend of new cases of leprosy in children under 15 years of age in the state of Bahia, Brazil, between 2007 and 2017	In the state of Bahia, northeast Brazil, between 2007 and 2017	2,298	844	37	**1,454**	63.27	9/9
39	Freitas et al. (2017)	Sociodemographic, clinical and epidemiological profile of leprosy in children under 15 years of age, Mato Grosso, Brazil (90)	Cross-sectional epidemiological	LILACS	Characterize the sociodemographic, clinical and epidemiological profile of leprosy in children under 15 years of age registered in Mato Grosso, from 2001 to 2013	Mato Grosso, Midwest (2001–2013)	2,455	722	29.40	**1,652**	67.30	9/9
40	De Camargo et al. (2018)	Polymorphisms in the TGFB1 and IL2RA genes are associated with clinical forms of leprosy in Brazilian population (91)	Cross-sectional and descriptive	SciELO, PUBMED	We performed an association study for IL2RA and TGFB! genes with clinical forms of leprosy based on two case–control samples. These scenes encode molecules important for the immunosuppressive activity of Trea cells and present differential expressions according to the clinical forms of leprosy. Furthermore. IL2RA is a positional candidate gene because it is located close to the region of chromosome 10o13. presenting a link to PB leprosy	Rondonópolis/Mato Grosso/Center-West, São Paulo/southeast	885	690	77.97	**195**	22.03	9/10
41	Pacheco et al. (2014)	Prevalence and control of leprosy: research in an urban settlement in São Luís, Maranhão, Brazil (92)	Descriptive retrospective	LILACS	To investigate the prevalence of disabilities in patients with Leprosy in three Basic Health Units (UBS) in São Luís in Maranhão and preliminarily discuss possible ways to control the disease.	Urban occupation of São Luís, Maranhão, Brazil 2008–2009	24	21	87.5	**3**	12.5	6/8
42	De Sousa et al. (2021)	Reviewing the therapeutic management of leprosy in primary care: demand case series referred to a University Hospital in the Midwest region of Brazil (93)	Analytical retrospective	SciELO, PUBMED	To analyze the therapeutic management of leprosy patients referred from primary health services to a specialized service.	University Hospital of the Midwest region of Brazil (2016–2017)	225	210	93.3	**14**	6.22	9/9
43	Goulart et al. (2008)	Risk and protective factors for leprosy development determined by epidemiological surveillance of household contacts (57)	Descriptive and cross-sectional	Pubmed	Establish the relative risks or preventive effects of the presence of BCG vaccination, the Mitsuda test and the ML-Flow assay.	Hospital das Clínicas of the Federal University of Uberlândia, Uberlândia, Minas Gerais, Southeast (2002–2007)	28	8	28.6	**20**	71.40	6/8
44	Oliveira et al. (2020)	Serological evidence of Toxoplasma gondii infection as potential risk for the development of lepromatous leprosy in an endemic area for both neglected tropical diseases in Brazil (94)	Descriptive analytical	BMC	Investigate the possible influence of *M. leprae*/T. gondii in the manifestation of leprosy and its clinical forms.	City of Campos dos Goytacazes, state of Rio de Janeiro, Southeast (2015–2019)	291	136	46.7	**63**	21.6	10/10
45	Ramos et al. (2021)	Social inequalities and their association with the leprosy burden in a Brazilian city of low endemicity: An ecological study (95)	Ecological	Pubmed	To analyze the association between social inequalities and leprosy burden in a low endemicity scenario in the state of São Paulo, Brazil.	Municipality of Ribeirão Preto, state of São Paulo, southeast, Brazil, 2006–2026	608	482	79.28	**126**	20.72	7/9
46	Barbosa et al. (2020)	Spatial analysis of epidemiological and quality indicators of health services for leprosy in hyperendemic areas in Northeastern Brazil (96)	Ecological	Pubmed, SciELO	Analyze spatial patterns through epidemiological indicators	Pernambuco, Northeast, Brazil 2005–2014	28,895	14,109	49	**14,740**	51	9/9
47	Nicchio et al. (2016)	Spatial and temporal epidemiology of *Mycobacterium leprae* infection among leprosy patients and household contacts of an endemic region in Southeast Brazil (60)	Epidemiological	Science DIRECT, SciELO	Objective of evaluating the distribution pattern of the disease and the areas at greatest risk of illness, in a highly endemic municipality (Ituiutaba, MG) in the southeast region of Brazil	Municipality of Ituiutaba, in the state of Minas Gerais located in southeastern Brazil, 2004–2014	371	213	57.40	**158**	42.60	8/9
48	Dos Santos et al. (2020)	Study of TNF- α, IFN- γ, TGF- β, IL-6, and IL-10 gene polymorphism in individuals from the leprosy endemic area in the Brazilian Amazon (97)	Cross-sectional	ScienceDirect	Verify the relationship between TNF-ÿ rs1800629 cytokine polymorphisms; IFN-ÿ rs2430561; TGF-ÿ rs1982073 and rs1800471; IL-6 rs180079 and IL-10 rs1800896, rs1800871 and rs1800872 leprosy	Curionopolis, Goianésia do Pará, Canaã dos Carajás, North, Brazil between January and March 2016	106	28	26.42	**24**	22.64	6/8

The absolute frequencies of paucibacillary cases of the disease in each study were kept in bold.

### 3.3 Results of meta-analysis and about the publication bias

**Figure 3:**
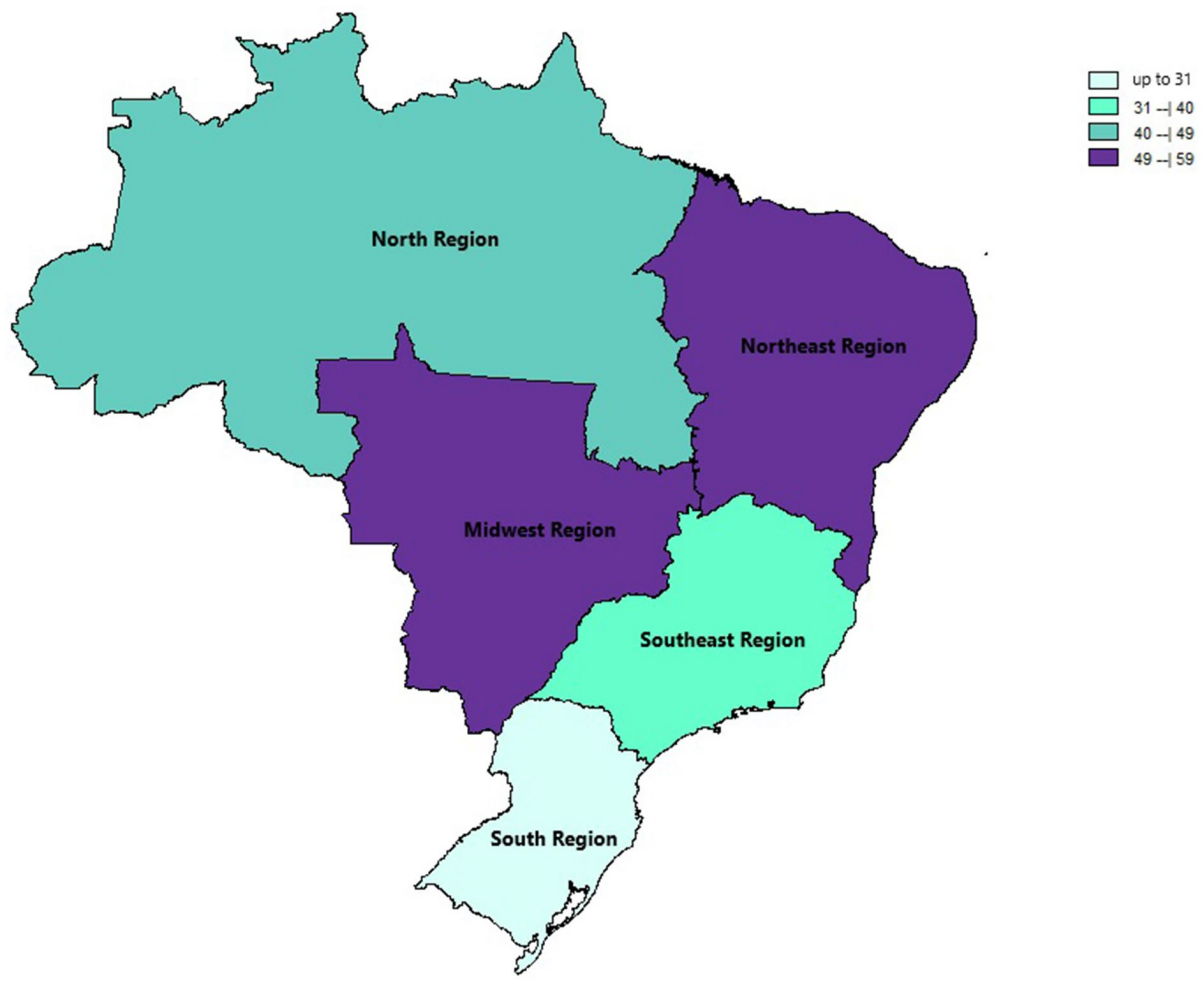
Thematic map of the estimated percentage value (%) of paucibacillary cases with national distribution over the years of this review based on official regional groupings.

**Figure 4:**
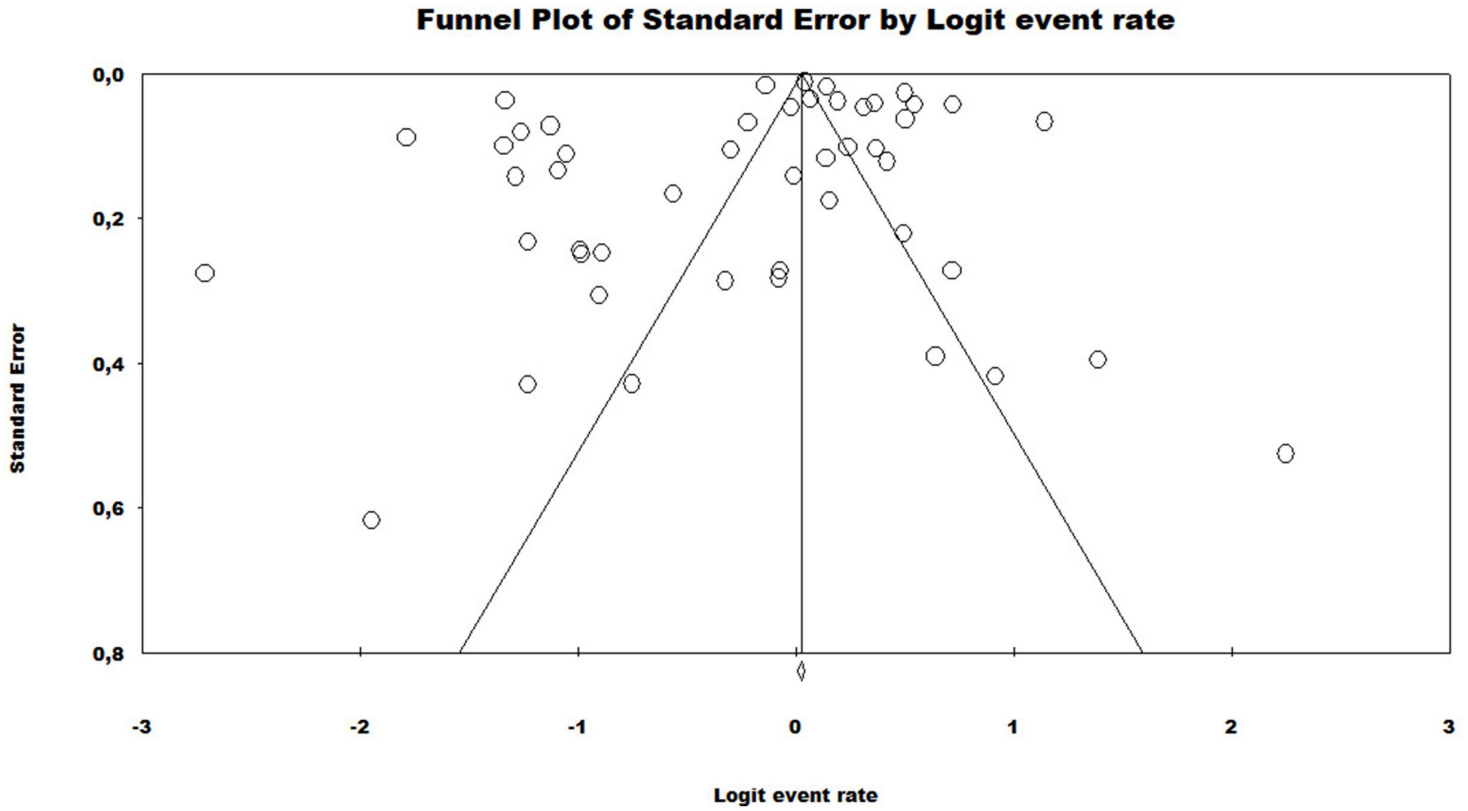
Funnel plot to assess heterogeneity between the studies included in the meta-analysis. Included studies are represented by circles, which should be evenly distributed around the overall effect to resemble an inverted funnel. The narrowest area of the funnel corresponds to the most precise studies, located closest to the real value. The standard error, displayed on the Y axis of the graph as a measure of dispersion, is influenced by the sample size of each study.

In the generalized analysis of 48 studies, the paucibacillary prevalence rate was 50.5% or 0.505 (95% CI = 0.502–0.509). The disparities between the analyzes showed statistically significant differences (*Q*-value 4302.681; df 47; I^2^ 98.905), meaning that the results had considerable variance between the studies, with heterogeneity evidenced by the high value of I^2^ (98.905). This analysis demonstrated the Midwest region, with a frequency of 58.4% or 0.584 (95% CI = 0.569–0.599), the Northeast region 53.6% or 0.536 (95% CI = 0.532–0.540), the North region 48.2% or 0.482 (95% CI = 0.474–0.489), Southeast region 39.6% or 0.396 (95% CI = 0.383–0.409), South region 21.4% or 0.214 (95% CI = 0.202–0.226) and finally, a study evaluated two regions (Midwest and Southeast) with 22% (95% CI = 0.194–0.249). A thematic map of these estimated percentage values in Brazilian regions was characterized in Figure 3. Despite the asymmetric distribution of the funnel graph (Figure 4), as it is a proportion-type meta-analysis, when applying the Begg (Kendall Tau *p* = 0.35) and Egger (*p* = 0.20) tests resulted in low publication bias.

### 3.4 Meta-regression of studies included by year of publication

**Figure 5:**
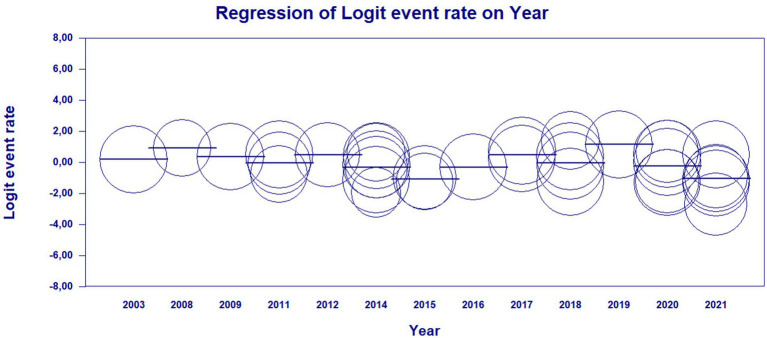
Scatterplot of meta-regression of event rate by covariate, year of study publication. Bubble size is inversely associated with study variance. The solid line reflects the linear regression.

**Figure 6:**
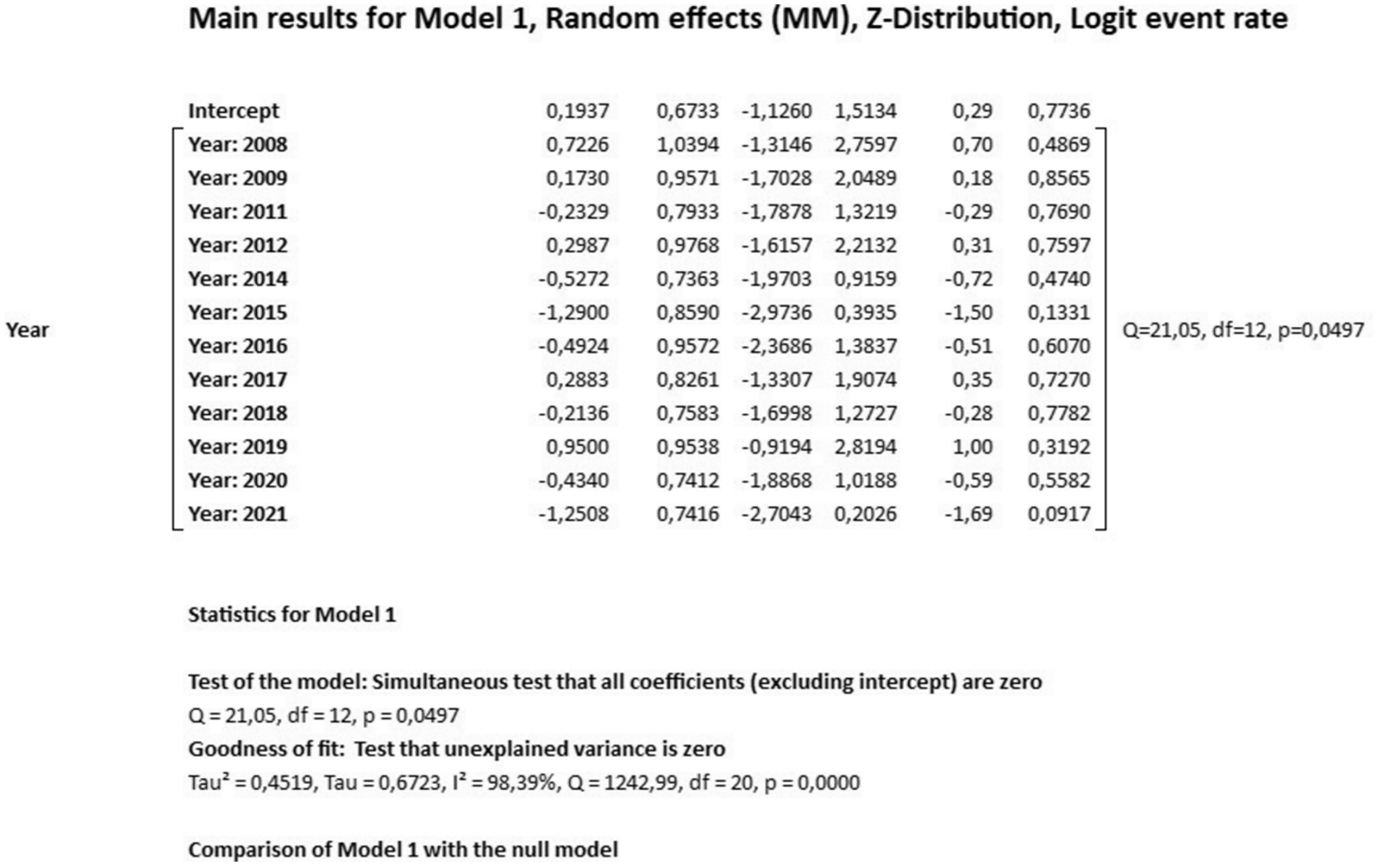
Main results for model, randomized effect, Z distribution and logit event rate.

The results of the meta-regression when using the moderator year of publication of the studies to verify the origin of the bias, the results showed that it had a significant association with the presence of heterogeneity in the findings of the average rates over the years (*Q* = 21.05; df = 12; *p* = 0.0497). The possible origin of the high heterogeneity of the data presented in the meta-analysis was found with the moderator “year of publication” in the years 2010 and 2023, indicating that there may be specific factors associated with these years of studies detecting leprosy in the Brazilian population that influence the high heterogeneity found (Figures 5, 6).

## 4 Discussion

This study is among the first systematic attempts to estimate the prevalence of paucibacillary cases in Brazil in the last 20 years. Forty eight articles published between 2003 and October 2023 were analyzed. No systematic review studies on the prevalence of paucibacillary cases in Brazil were identified. In this study, the prevalence of paucibacillary cases was estimated at 50.5%, with a 95% confidence interval ranging from 50.2 to 50.9%, which indicates a relatively close consistency in the proportion of PB leprosy cases in the studies analyzed, with this prevalence being within the specific range and contributing to the understanding of the distribution of PB cases in the studied population. This variation can be influenced by several factors, such as differences in geographic regions, populations analyzed, ages analyzed, data collection methods, among others (20, 21).

The measure of identifying leprosy early is crucial in preventing the transmission of the disease, as well as avoiding the most serious forms of neural disabilities, contributing to the achievement of the global strategy developed by the WHO that aims to interrupt transmission and eliminate cases, which indigenous people with a zero leprosy incidence rate (3, 22).

Research indicates that Brazil exhibits a diverse distribution of leprosy cases, with varying proportions of MB and PB classifications across different regions (23–26). For instance, a study highlighted that only 28% of cases were classified as paucibacillary, indicating a predominance of multibacillary cases (27). This suggests that the prevalence of PB cases is relatively low, particularly in hyperendemic areas. Therefore, this present meta-analysis does not align with the pre-existing literature, considering that only the South Region (characterized by having states with low endemicity) among all the other analyses had a percentage similar to that previously recorded (28). The causes behind these discrepancies in the detection rate of this meta-analysis in comparison with other Brazilian studies already published may arise, for example, from a methodological nature, considering that this systematic review encompassed a broader range of studies over a 20-year period, potentially capturing more recent data and variations in reporting practices. The inclusion of diverse epidemiological study designs (case–control, cohort, cross-sectional) may have provided a more comprehensive picture of leprosy epidemiology. The heightened awareness and improved diagnostic capabilities in recent years may have led to more cases being identified as PB, especially in areas previously underreported. This could explain the higher prevalence rate compared to earlier studies (29).

When comparing the results of this meta-analysis with studies from other countries with similar leprosy burdens, several patterns emerge. In case of India, the country with the highest leprosy burden, it has reported varying prevalence rates of PB cases, often around 50% in certain regions. This similarity suggests that Brazil’s epidemiological landscape may reflect broader trends observed in high-burden countries (30). In countries like Nigeria and Mozambique, studies have indicated that PB cases comprise a significant portion of reported leprosy cases, often due to similar socioeconomic and healthcare challenges (31, 32). The prevalence rates can be comparable to those found in Brazil, highlighting the need for targeted public health strategies. Countries such as Indonesia and Myanmar have shown similar trends in PB prevalence, often influenced by local healthcare infrastructure and public health initiatives. The importance of early detection and treatment in reducing the burden of leprosy is a common theme across these regions (33, 34).

Brazil is still a country with a high leprosy endemicity, with a prevalence >1 per 10,000 inhabitants, representing around 94% of cases among Latin American countries. The epidemiological bulletins show the detection of new cases regarding the clinical form, a greater frequency in the prevalence coefficients of multibacillary cases, with the year 2022 representing 80.2% MB, other studies also brought results similar to the predominance of MB cases, showing late detection and active transmission of leprosy. In 2020 there was a significant reduction in new cases of leprosy compared to 2019, this decrease is probably related to the reduction in detection during the COVID-19 pandemic that affected health services around the world (35–37).Furthermore, a meta-analysis that obtained a high proportion of MB cases in several countries, including Brazil, highlighted the importance of improving the monitoring and treatment of individuals at risk of developing the most severe forms (38). The active search for contacts in these cases should be an important measure for early diagnosis, with household contacts being crucial, since there is a greater risk of developing the disease due to prolonged exposure to untreated patients. However, there is still a lack of studies regarding asymptomatic *M. leprae* infection, and more research is needed on this topic to facilitate diagnosis in the early stages and detect individuals infected by the bacillus (39).

As it is a slowly evolving disease, there is still no gold standard for diagnosing leprosy. A study that investigated the effectiveness of qPCR in diagnosing PB reports the difficulty and challenges that even experienced specialists encounter in interpreting the clinical differential diagnosis. and histopathological in PB cases, which may be one of the factors responsible for numerous late diagnoses of the disease (12).

Butlin and Lockwood, in a study that evaluated changes in the proportions of PB leprosy cases using global data published by the WHO in a period from 1985 to 2017, demonstrated the continuous decrease in PB cases over the years, in which until 2003 the paucibacillary form was more frequent than the multibacillary clinical form, which may be linked to significant changes in the correct classification of the patient, since therapeutic protocols and guidelines recommend that in case of doubts about the PB and MB operational classification, it can be classified as multibacillary (40). The authors highlight the need to improve the accuracy of the categorization of clinical forms, with more investigations being necessary in the validation of operational forms by national control programs (3).

When analyzing the Brazilian regions, it demonstrated that the Midwest region had the highest frequency (58.4%), followed by the Northeast (53.6%), North (48.2%), Southeast (39.6%) regions. and finally, the South region (21.4%). Similar data were found in an epidemiological study from 2005 to 2015, which obtained the highest leprosy prevalence coefficients in the Midwest, North and Northeast regions, being related to regional socioeconomic inequalities, regarding the development and quality of life of the population between regions of Brazil (41, 42).

This variation in the prevalence of paucibacillary infections highlights the Midwest region with the highest rate, while the South region has a notably lower rate, highlighting disparities between regions that contribute to the persistence of the disease. The vast territorial extension of Brazil, socioeconomic inequalities between regions and the gap in tackling social determinants were identified as important factors responsible for this discrepancy, in which the economically less favored regions stand out as being the most endemic for leprosy (43, 44).

The geographical distribution of leprosy in Brazil is notably uneven, with higher rates of new case detection in the Northern, Northeastern, and Midwest regions. For example, the states of Mato Grosso and Tocantins have reported extremely high new case detection rates (NCDR) of 82.03 and 69.88 per 100,000 inhabitants, respectively (45). This spatial variation points to the influence of socio-economic factors, healthcare access, and environmental conditions that contribute to the epidemiology of leprosy.

The implications of this geographical heterogeneity are profound for subgroup analyses. It necessitates tailored public health interventions that consider local epidemiological patterns. For instance, high-risk areas may require intensified screening and treatment programs, while regions with lower incidence rates might focus on surveillance and education to prevent outbreaks (46). Moreover, understanding the factors that contribute to the higher prevalence of MB cases in certain regions can inform targeted strategies to address the underlying causes of transmission. As a measure of the patient’s capacity to eradicate bacillus, the ratio of multibacillary to paucibacillary instances represents transmission of leprosy (47).

For example, factors such as population density, socioeconomic status, and healthcare infrastructure can significantly affect the distribution of leprosy cases, influencing both detection and classification. Regions with higher levels of poverty often experience worse health outcomes, including higher rates of leprosy. The Northeast region, for instance, has historically faced economic challenges that correlate with increased disease prevalence (48). Lower educational attainment can lead to a lack of awareness about leprosy symptoms and transmission, resulting in delayed diagnosis and treatment. Educational disparities are often pronounced in poorer regions (49). Inadequate housing and sanitation are more prevalent in economically disadvantaged areas, facilitating the spread of infectious diseases like leprosy (50). Access to healthcare services is often limited in rural and impoverished areas. Regions with fewer healthcare facilities may struggle to provide timely diagnosis and treatment for leprosy, affecting the classification of cases as PB or MB (51). Furthermore, robust public health infrastructure and effective public health interventions are crucial for effective disease surveillance and response, so that national and local health policies that prioritize leprosy control can significantly impact prevalence rates (52).

The high heterogeneity presented in the meta-analysis can be justified by the differences and variability in the characteristics of the studies, which may be related to inadequate study design, limitation of the number of samples and also the weakness in the standardization of controls. The meta-regression analyzes showed a significant association between the year of publication of the studies and the heterogeneity of the results, highlighting the years 2010 and 2023 with periods of possible specific influences, which highlights the importance of more studies to investigate the temporality of the leprosy.

Among the studies analyzed in this meta-analysis, an increase in paucibacillary cases was observed in studies that investigated the population under 15 years of age. It is known that detection rates in this age group indicate recent infection, resulting in active transmission of *M. leprae* between contacts, with early detection especially in endemic locations having significant relevance (53–58). An incompleteness of information was observed in some studies, in which PB and MB data did not correspond to the total number of diagnosed cases, with the total number of diagnosed cases being greater than the sum of PB and MB data, with this information in the studies being represented as “ignored” or “not filled in.” Completing the information is essential for understanding the real epidemiological situation so that actions related to the disease can be planned (59, 60).

The limitations of this study included (I) the search methodology carried out, through the specific descriptors applied; (II) the high heterogeneity of the studies, probably justified due to the period of the studies, differences in the quality of the data, which may have affected the precision of the results; (III) possible information and selection biases, sample representativeness; and (IV) there may be omissions in the included studies.

More studies are needed to investigate the prevalence of the paucibacillary operational classification, which may be crucial for reducing transmission, with treatment of paucibacillary as it has a low bacillary load, and early diagnosis to prevent worsening due to late diagnosis of leprosy. and greater community transmission. The quality of paucibacillary diagnosis and treatment can influence the reduction of active transmission, as it will prevent the evolution of low numbers of bacilli to high bacilli, the multibacillary which is the most important in the transmission of leprosy, so improving paucibacillary surveillance is a strategy effective in reducing the incidence of leprosy, and achieving the goals established by WHO and MS agreements in the coming years in the country (61).

## 5 Conclusion

This systematic review and meta-analysis provided comprehensive information on the prevalence of paucibacillary cases in Brazil from 2003 to 2023, using data from 48 published studies. The average prevalence rate in the Brazilian population was estimated at 50.5%, which indicates a relatively homogeneous distribution of the PB form of leprosy in the sample studies. The analysis of paucibacillary cases in Brazil is marked by significant geographical heterogeneity, which has critical implications for public health strategies. Addressing these disparities through targeted interventions and localized analyses can enhance the effectiveness of leprosy control efforts in the country. The ongoing monitoring of case classifications and regional trends will be essential in adapting strategies to combat this public health challenge effectively. In this sense, this research can contribute by filling the gap and providing new and relevant data for understanding the prevalence of paucibacillary leprosy in Brazil, in addition to providing important information for the formulation of social policies with public health strategies and guidance for future research on the theme.

## Data Availability

The original contributions presented in the study are included in the article/Supplementary material, further inquiries can be directed to the corresponding author.

## Footnotes

^1^ https://pubmed.ncbi.nlm.nih.gov/

^2^ https://www.scielo.br/

^3^ https://www.sciencedirect.com/

^4^ https://lilacs.bvsalud.org/
